# The Treatment of Whooping Cough

**Published:** 1888-07

**Authors:** Emory Lanphear

**Affiliations:** Kansas City, Mo., Professor of Diseases of the Mind and Nervous System, in the University of Kansas City


### THE TREATMENT OF WHOOPING COUGH.

*By Emory Lanphear, M. D., Kansas City, Mo., Professor of Diseases of the Mind and Nervous System, in the University of Kansas City.*

For Daniel's Texas Medical Journal.

ALTHOUGH bacteriologists claim that a specific germ of pertussis has been demonstrated (the "bacillus pertussis,"—a micro-organism discovered by Afanasieff—a microbe having properties sufficient to distinguish it from other bacteria,) therapeutic measures based upon this idea have not been, in my hands at least, as gratifying as would be anticipated from the claims of the enthusiastic supporters of the germ theory. Corrosive sublimate, permanganate of potassium, boric acid, thymol, the salicylates, carbolic acid, etc., have been, both locally and internally, "tried and found wanting," the only drug which might be classed as "antiseptic" which gave any satisfaction whatever, being quinine, used as a spray to the fauces, in conjunction with its internal administration; that the favorable results obtained from its use were due to any antiseptic properties, I am not prepared to concede.

From drugs given upon the theory that the disease might be of nervous origin, little better can be said: cannabis indica, hyoscyamus, belladonna, and all others of that class, have been of little avail; so, too, cocaine, either internally or as a spray—though my experience with it has been rather limited—has proven unsatisfactory. Bromides have, however, been extensively prescribed (because of their tranquilizing effect), and with considerable success; not in abating or curing the disease, but in modifying its severity. Upon the bromides, then, I have come to chiefly rely, though in many cases where there is difficulty in retaining nauseous medicines, I give with almost equally beneficial results:

| | | |
|---|---|---|
| ℞ | Codeinæ sulphatis | gr. j. |
| | Syrupi scillæ comp. | f. ʒ ij. |

Syrupi tolutani f. ʒ vi.

Syrupi pruni virgin. f. ℥ j.

Misce. Sig. One-half, to one teaspoonful every two or three hours for a child of two years of age.

All opiates other than codeine are preferably eschewed. Chloral is excellent in many cases, but I usually depend upon the above formula or the elixir of bromide of potassium.

Recently, however, because of its wonderful effect in controlling nervous pain, like migraine, neuralgia, etc., and its surprising sedative properties, I have resorted to antipyrine with, thus far, the best results derived from any one drug, or combination of drugs. I do not regard it as curative—in fact nothing is—but it does control the severity of the paroxysms to a gratifying extent. For a child of two years, I write:

℞ Antipyrin ʒ i.

Aq. menth. pip. q. s. ut. ft. solut.

Add:

Syrupi pruni Virg. ad. f℥ ij.

Misce. Sig. One teaspooful every six hours, continued throughout the entire course of the affection if indicated.

The bromide mixture may be given between each of these doses, with advantage in some cases.

Of course the greatest care must be taken to insure proper diet and the best hygienic surroundings, for upon these, coupled with sustaining agents, may depend the life of the little patient.

